# DNA Break Site at Fragile Subtelomeres Determines Probability and Mechanism of Antigenic Variation in African Trypanosomes

**DOI:** 10.1371/journal.ppat.1003260

**Published:** 2013-03-28

**Authors:** Lucy Glover, Sam Alsford, David Horn

**Affiliations:** London School of Hygiene & Tropical Medicine, London, United Kingdom; University of California, Los Angeles, United States of America

## Abstract

Antigenic variation in African trypanosomes requires monoallelic transcription and switching of variant surface glycoprotein (*VSG*) genes. The transcribed *VSG*, always flanked by ‘70 bp’-repeats and telomeric-repeats, is either replaced through DNA double-strand break (DSB) repair or transcriptionally inactivated. However, little is known about the subtelomeric DSBs that naturally trigger antigenic variation in *Trypanosoma brucei*, the subsequent DNA damage responses, or how these responses determine the mechanism of *VSG* switching. We found that DSBs naturally accumulate close to both transcribed and non-transcribed telomeres. We then induced high-efficiency meganuclease-mediated DSBs and monitored DSB-responses and DSB-survivors. By inducing breaks at distinct sites within both transcribed and silent *VSG* transcription units and assessing local DNA resection, histone modification, G_2_/M-checkpoint activation, and both RAD51-dependent and independent repair, we reveal how breaks at different sites trigger distinct responses and, in ‘active-site’ survivors, different switching mechanisms. At the active site, we find that promoter-adjacent breaks typically failed to trigger switching, 70 bp-repeat-adjacent breaks almost always triggered switching through 70 bp-repeat recombination (∼60% RAD51-dependent), and telomere-repeat-adjacent breaks triggered switching through loss of the *VSG* expression site (25% of survivors). Expression site loss was associated with G_2_/M-checkpoint bypass, while 70 bp-repeat-recombination was associated with DNA-resection, γH2A-focus assembly and a G_2_/M-checkpoint. Thus, the probability and mechanism of antigenic switching are highly dependent upon the location of the break. We conclude that 70 bp-repeat-adjacent and telomere-repeat-adjacent breaks trigger distinct checkpoint responses and *VSG* switching pathways. Our results show how subtelomere fragility can generate the triggers for the major antigenic variation mechanisms in the African trypanosome.

## Author Summary

Previous studies on antigenic variation in African trypanosomes relied upon positive or negative selection, yielding only cells that underwent variation. This made it difficult to define individual switched clones as independent, potentially introduced bias in the relative contribution of each switching mechanism and precluded analysis of cells undergoing switching. We show that DNA double-strand breaks (DSBs) naturally accumulate close to *Trypanosoma brucei* telomeres. Using the I-*Sce*I meganuclease, we then established a system to trigger breaks in all cells in a population. The specificity, temporal constraint and efficiency of cleavage facilitated the application of a quantitative approach to dissecting subtelomeric break responses and their consequences. Accordingly, we show that the DSB-site determines probability and mechanism of antigenic switching, that DSBs can trigger switching *via* recombination or transcription inactivation and that a checkpoint-bypass mechanism can explain switching via *VSG* expression site deletion. Our results provide major new insights into the mechanisms underlying antigenic variation and provide a new model to explain how the repeats flanking *VSG* genes serve distinct roles in fragility and recombination. The findings are also relevant to telomeric gene rearrangements that control immune evasion in other protozoal, fungal and bacterial pathogens such as *Plasmodium*, *Pneumocystis* and *Borrelia* species, respectively.

## Introduction

Several important parasites, including those that cause malaria and Human African Trypanosomiasis (HAT), achieve antigenic variation and evasion of the host adaptive immune response through monoallelic expression and clonal phenotypic variation of surface proteins [1], [2]. The African trypanosomes are flagellated parasitic protists of major medical and veterinary importance. They are the causative agents of HAT, and Nagana in cattle, and they proliferate in the mammalian host bloodstream. In *Trypanosoma brucei*, antigenic variation requires mono-telomeric expression and switching of variant surface glycoprotein genes (*VSGs*). It is this continuous process of allelic exclusion, transcription of only one telomeric *VSG* at a time in each cell, which is essential for the persistence of a chronic infection. *T. brucei* has long been a paradigm for antigenic variation but the molecular triggers and the mechanisms mediating *VSG* recombination and switching are not fully understood.

Telomeres are specialized structures that cap chromosome ends, consisting of long tracts of T_2_AG_3_-repeats in *T. brucei* and in human cells. *T. brucei* subtelomeres are the exclusive expression sites (ESs) for *VSG* genes [3]. One among approximately fifteen bloodstream-form ESs (BESs) is active in each cell and RNA polymerase I drives transcription at an extra-nucleolar site known as the expression site body (ESB) [4], [5], [6]. The BESs are polycistronic transcription units with promoters located up to 60 kbp from the telomere-adjacent *VSG* [7]. Sequencing of multiple BESs revealed a conserved arrangement, with *VSGs* flanked by repetitive sequences; the telomeric repeats (up to 15 kbp tracts) downstream and the 70-bp repeats (0.2–7.1 kbp tracts) upstream [7]. The minichromosomes, of which there are up to 100 copies per genome, contain additional archival, non-transcribed *VSG* genes flanked by telomeric repeats and 70-bp repeats. The BESs typically also encode several Expression Site Associated Genes (ESAGs), but these genes are always separated from the *VSG* by 70-bp repeats [7]. The single active, transcribed *VSG* accounts for approximately one-tenth of total cell protein, which forms a dense protective coat on each cell [8], while inactive *VSG* mRNAs are approximately 10,000-fold less abundant than the active *VSG* mRNA [9].

Antigenic variation appears to be a stochastic process, typically involving duplicative transposition and replacement of the active *VSG* [10], [11]. The process can also occur via loss or replacement of the entire active BES [12], [13], [14], [15] or via *in situ* BES switching, whereby activation of a previously silent BES is coordinated with BES inactivation, typically with no detected DNA rearrangement. The majority of archival *VSGs*, up to 2,000 subtelomeric genes and pseudogenes [16], [17], are not associated with BES promoters. Thus, recombination and replacement of the active *VSG* is required to utilize this archive for long-term immune evasion. 70-bp repeat sequences define the 5′ boundaries for *VSG* recombination [18] and 70-bp repeats are found upstream of most archival *VSG*s [17], serving as potential templates for homologous recombination; this involves gene conversion or, in the case of telomeric *VSGs*, break-induced replication (BIR), whereby the template is copied to the chromosome end [10]. The long 70-bp repeat tracts found at active BESs are, therefore, recombination substrates that facilitate the translocation of archival *VSG* genes to the transcribed telomere [19]. It has been proposed that this transcribed 70-bp repeat tract is also fragile, such that the DNA breaks that trigger antigenic variation originate here [10].

The dominant mechanism of chromosomal double-strand break (DSB) repair in *T. brucei* is homologous recombination [20]. RAD51-independent, microhomology-mediated end-joining (MMEJ) also operates, while non-homologous end-joining has not been detected [21]. Studies on strains lacking the RAD51 homologous strand-exchange protein [22], the RAD51-3 paralogue [23] or the RAD51-interacting protein, BRCA2 [24], indicate that each of these factors promotes *VSG* switching. In contrast, TOPO3α, a type 1A topoisomerase, functions with RMI1 as an anti-recombinase, suppressing BES crossovers but promoting duplicative *VSG* transposition through 70-bp repeat recombination [13], [14].

Despite recent progress, little is known about the subtelomeric DSBs that naturally trigger antigenic variation in *T. brucei*, the subsequent DNA damage responses, or how these responses determine the mechanism of *VSG* switching. We show that natural breaks accumulate close to the telomere in both transcribed and non-transcribed BESs. We induced DSBs at different sites within both active and silent BESs and recovered survivors for analysis, those that switch and those that don't. We find that the site of the DSB has a major impact on the DSB response and the probability and mechanism of *VSG* switching.

## Results

### *T. brucei* Subtelomeres Are Fragile Sites

**Figure 1:**
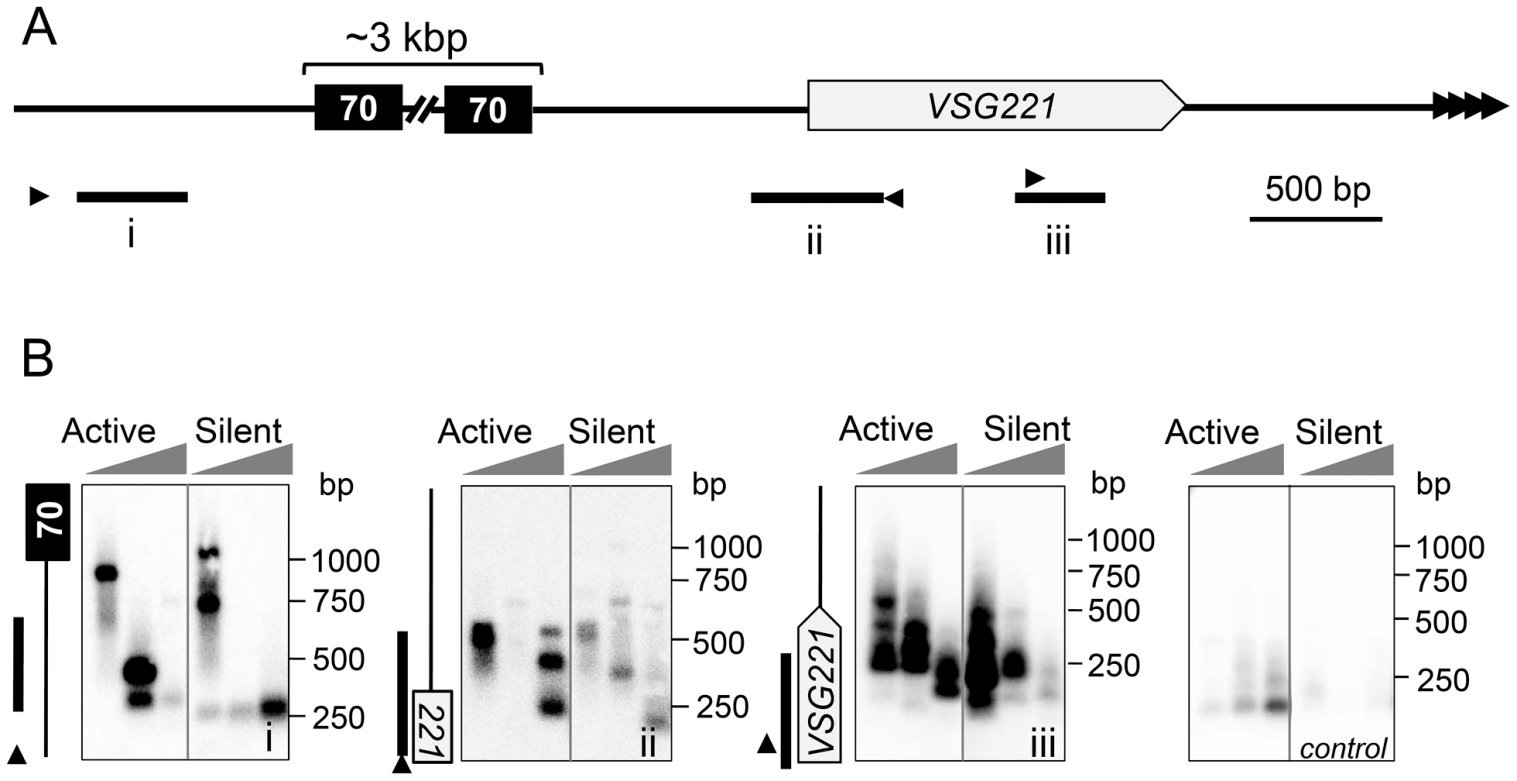
*T. brucei* subtelomeres are fragile sites. (A) The schematic shows the subtelomeric *VSG221* (also known as *VSG2*) locus and indicates the locations (i–iii) of the specific LM-PCR primers (arrowheads) and probes (black bars). 70, 70-bp repeats. (B) Southern blotting of LM-PCR products derived from both the active and silent BES. A chromosome internal primer-probe combination was used as a control (Tb11.02.2110 locus). Grey triangles represent increasing number of cells surveyed in each PCR reaction (4,000, 20,000 and 100,000). The LM-PCR approach is prone to a loss of signal as the number of cells in the sample is increased.

Although artificial DNA breaks between the *VSG* and the 70-bp repeats at the active BES enhance antigenic variation in *T. brucei*, the presence of natural breaks has only been mapped to the *VSG*-distal side of these repeats [10]. We, therefore, used ligation-mediated PCR (LM-PCR) to investigate the distribution of natural DSBs in the vicinity of the *VSG221* gene, in either the active transcribed or silent state; the *VSG221* locus on chromosome 6a is single-copy and hemizygous. LM-PCR involves the ligation of a specific oligonucleotide to sites of DSBs followed by amplification of products using primers specific for the ligated oligonucleotide and for the locus of interest. The PCR products, each representing a distinct DSB, are then separated on a gel and detected using an appropriate probe. LM-PCR, therefore, provides a ‘snap-shot’ of DSBs in a population of cells. We used three specific *VSG221* BES primer-probe combinations to assay breaks across three distinct regions (FIG. 1A; see maps to the left-hand side of the blots in FIG. 1B). A chromosome-internal primer-probe combination was used as a control. LM-PCR assays revealed DSBs in all three subtelomeric regions and, in contrast to a previous report [10], transcription status had little impact on the number of DSBs, which were detected at a similar frequency regardless of whether the *VSG* was transcribed or silent (FIG. 1B). Thus, we suggest that DNA replication rather than transcription generates natural breaks.

Following a comparison of the subtelomeric regions examined, we tentatively suggest that breaks could be more frequent closer to the telomere. We detected several *VSG221*-flanking breaks when only 4,000 cells were sampled (FIG. 1B), meaning that the frequency of these potential antigenic variation triggers exceeds the frequency of antigenic variation by two orders of magnitude; variants arise at a rate of approximately 1×10^−5^ per cell division [13]. We conclude that natural subtelomeric breaks typically fail to trigger antigenic variation.

### DNA Double-Strand Breaks at an Active *VSG* Expression Site Are Typically Lethal

**Figure 2:**
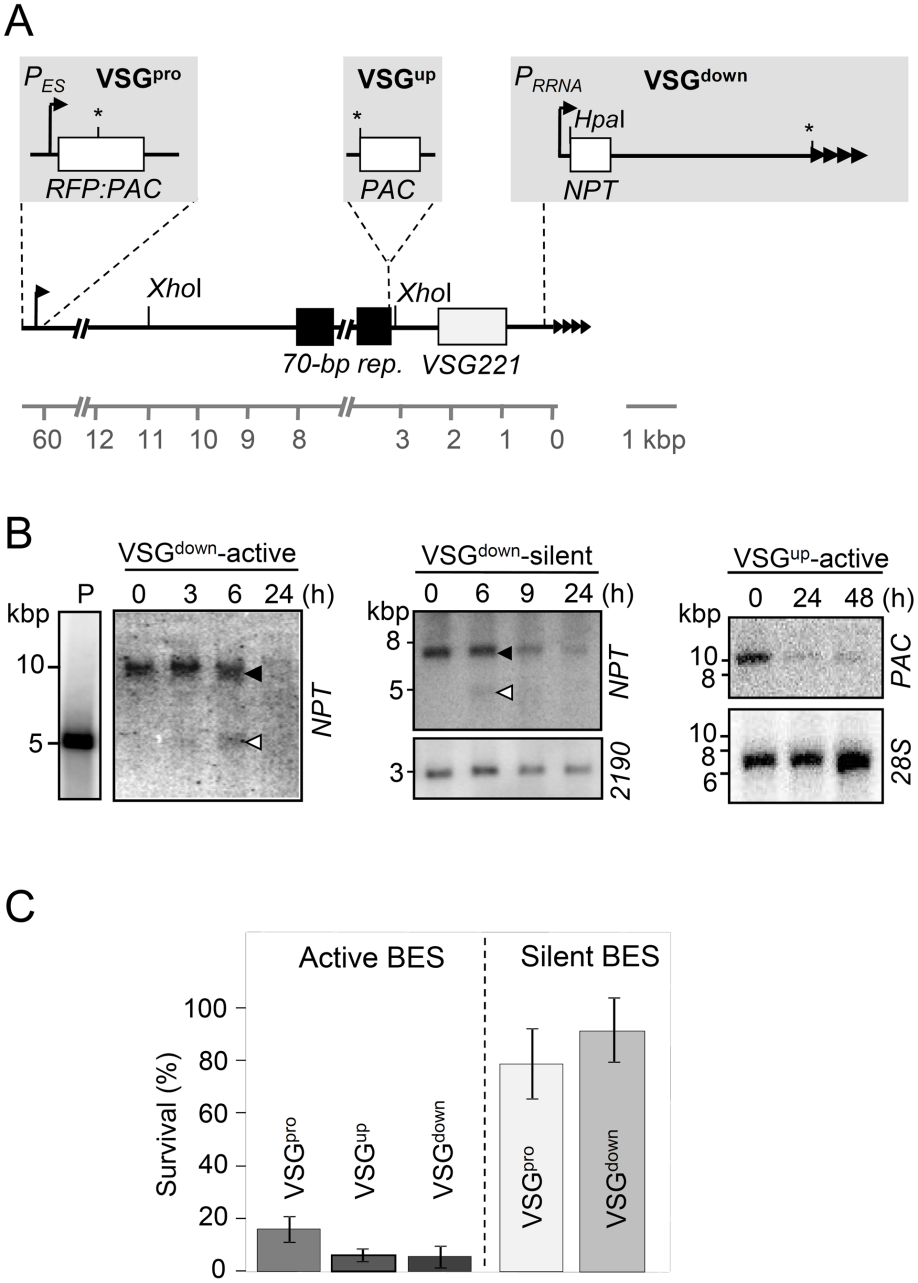
DNA double-strand breaks at an active *VSG* expression site are often lethal. (A) The schematic shows the *VSG221* BES on chromosome 6a with the I-*Sce*I sites (*) and reporters (*NPT*, Neomycin PhosphoTransferase; *RFP-PAC*, Red Fluorescent Protein-Puromycin *N-*ACetyltransferase; and *PAC*) incorporated using pESP-*R^S^P*, pES-70 or pTMF-Sce to give VSG^pro^, VSG^up^ or VSG^down^, strains respectively. Relevant restriction sites are shown. *P_ES_*, BES promoter; *P_RRNA_*, ribosomal RNA promoter (allows for low-level *NPT* transcription when the BES is ‘silent’). (B) DSB-induction is rapid and efficient. I-*Sce*I expression was induced with tetracycline (1 µg/ml), genomic DNA was extracted after different periods of time and digested with *Hpa*I (VSG^down^ strains) or *Xho*I (VSG^up^ strain); a plasmid control (P) was digested with *Hpa*I/I-*Sce*I. The probes are indicated and blots were rehybridized with either a ‘*2190*’ probe or a *28S* probe to show loading. Terminal restriction fragments (closed arrowheads) and *in vivo* cleaved fragments (open arrowheads) are indicated. (C) Clonogenic assays. Cells in which I-*Sce*I expression was induced were distributed in 96-well plates. Survivors were assessed after 7 days. Error bars, SD from three or more 96-well plates.

To examine the consequences of DSBs within BESs, a panel of *T. brucei* strains were established with a tetracycline-inducible I-*Sce*I meganuclease gene [20] and a single I-*Sce*I cleavage site within the active or silent *VSG221* BES; I-*Sce*I cleaves a specific 18-bp sequence and produces a single DSB. The three sites selected for integration of the I-*Sce*I site within the active *VSG221* BES (FIG. 2A) were adjacent to the BES promoter, approximately 60-kbp from the *VSG* (VSG^pro^); adjacent to the 70-bp repeats, upstream of the *VSG* (VSG^up^) or; adjacent to the T_2_AG_3_ repeats, downstream of the *VSG* (VSG^down^). Antigenic variation is not expected following recombination and repair at a silent site, but we did want to assess the impact of transcription on DSB repair. For this purpose, we also analyzed equivalent DSBs in VSG^pro^ and VSG^down^ strains with a silent *VSG221* BES. Immunofluorescence analysis confirmed that >99% of cells expressed VSG221 in the ‘active-VSG221’ strains and that <0.1% of cells expressed VSG221 in the ‘silent-VSG221’ strains. We also demonstrated that the latter strains could reactivate the *VSG221* BES (data not shown).

Using a combination of Southern blotting (FIG. 2B), PCR and drug-sensitivity assays for loss of expression of the break-adjacent selectable marker (data not shown, see FIG. 2A), we confirmed efficient and tightly regulated DSB-induction at the correct locus in all five strains detailed above; at least two independent assays used for each strain. The Southern blot analysis shown in Figure 2B reveals the terminal restriction fragments and the expected *in vivo* cleaved fragments in the active transcribed and silent VSG^down^ strains after 6 h of induction. Cleavage is almost complete after 24 h, as indicated by loss of the terminal restriction fragments, and we obtained similar results for the active VSG^up^ strain (FIG. 2B). In contrast, an I-*Sce*I site embedded within T_2_AG_3_-repeats was inaccessible (FIG. S1).

We next used a clonogenic assay to assess survival following DSBs in active and silent BESs. Cells were distributed in multi-well plates under DSB-inducing conditions and, after several days, wells with live cells were counted. Cloning efficiency averaged approximately 85% in cells with DSBs in the silent BES but was strikingly lower following DSBs in the active BES (FIG. 2C); only approximately 5% of VSG^up^ or VSG^down^ cells survived. The low cloning efficiency indicates that a break at the active BES is typically lethal. This may be because transcription interferes with the DSB response or, since VSG expression is required for cell-cycle progression [25], because the DSB response interferes with *VSG* transcription; the DSB response does indeed interfere with transcription in mouse cells [26]. Importantly, failure to tolerate a DSB is consistent with our observation that natural DSBs far exceed instances of antigenic variation (see above). We suggest that these natural DSBs at the active BES are also typically lethal.

### The Probability of Antigenic Variation Is Highly Dependent upon Subtelomeric Break Site

To explore antigenic variation following DSBs at the active transcribed *VSG* locus, we generated cloned DSB-survivors from the VSG^pro^ (24 clones), VSG^up^ (22 clones) and VSG^down^ (32 clones) strains. As above, the *VSG221* BES was maintained in the transcribed state prior to DSB-induction, using antibiotic-selection (see FIG. 2A), which was removed immediately prior to limiting dilution cloning under DSB-inducing conditions. This ensured that each cloned survivor represented an independent DSB-repair event and, unlike previous approaches, did not require any selection for cells that had modified expression of the *VSG* or a BES-reporter.

**Figure 3:**
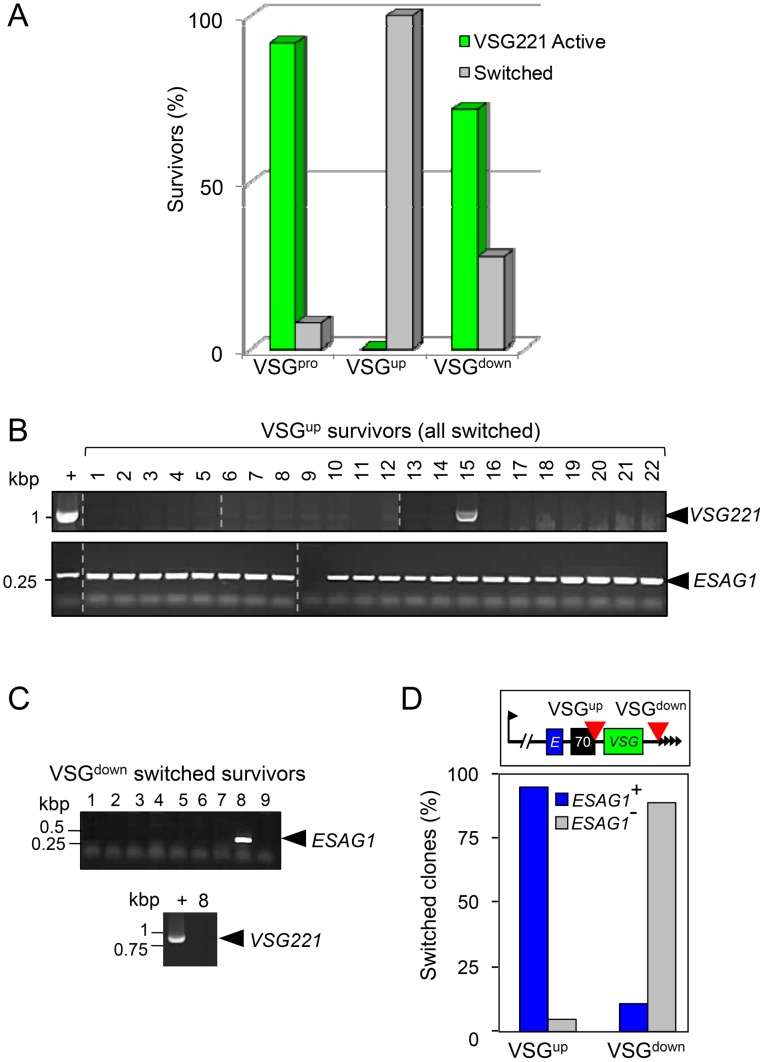
Probability and mechanism of antigenic variation are highly dependent upon subtelomeric break site. (A) DSB-induced survivors were assessed by *VSG221* immunofluorescence microscopy and scored as either VSG221 active or switched. VSG^pro^, n = 24; VSG^up^, n = 22; VSG^down^, n = 32. (B) PCR assays demonstrate *VSG221* and *ESAG1* gene status following I-*Sce*I-mediated cleavage in switched survivors from VSG^up^ cells. See the schematic maps in Figure 2A and Figure S2A for details. (C) As in B above but for VSG^down^ cells. (D) Comparison of *ESAG1* status of switched survivors from VSG^up^ (n = 22) and VSG^down^ strains (n = 9) as determined by PCR assay. The schematic shows the BES and DSB-sites, red arrowheads. *E*, *ESAG1*; 70, 70-bp repeats; *VSG*, *VSG221*.

**Figure 4:**
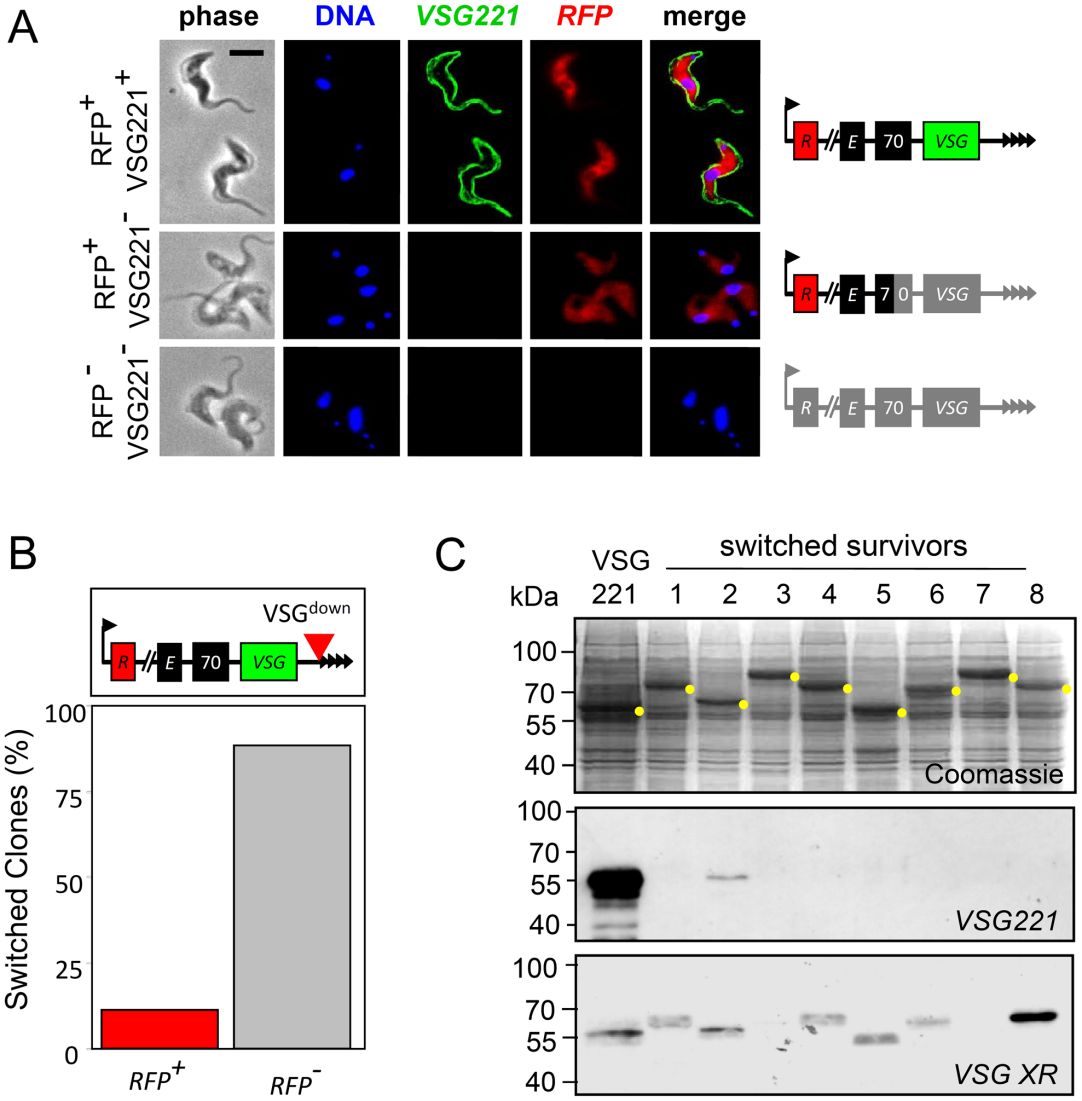
Telomere-adjacent breaks trigger BES loss or replacement. (A) Immunofluorescence analysis of VSG^down^ survivors. The schematic maps indicate the regions of the BES deleted/replaced in each case (grey) as determined using PCR assays (see FIG. 3B–C and FIG. S2). DNA was counter-stained with DAPI. Scale bar, 5 µm. (B) *RFP* status of switched survivors (n = 8) from the VSG^down^ strain, as determined by PCR assay (see FIG. S2C). Also see the schematic maps in Figure 2A and Figure S2A. (C) Examples of switched DSB-survivors. The Coomassie-stained gel indicates the abundant, clone-specific VSGs (yellow dots). The western blots were generated using a VSG221-specific or a VSG cross-reacting (VSG XR) antibody.

Using immunofluorescence analysis, we scored for survivors that had undergone antigenic variation (FIG. 3A; example fluorescence images are shown in FIG. 4A). In the VSG^pro^ strain, only two survivors (8%) had inactivated *VSG221*; in the VSG^up^ strain, all survivors (100%) had inactivated *VSG221*; and, in the VSG^down^ strain, nine survivors (28%) had inactivated *VSG221* (FIG. 3A). Thus, antigenic variation is efficiently triggered by a DSB adjacent to the 70-bp repeats, is less efficiently triggered by a DSB adjacent to the telomeric repeats and is rarely triggered by a DSB adjacent to the BES promoter. Antigenic variation in every DSB-survivor from the active VSG^up^ strain reflects a massive increase in switch frequency at 5×10^−2^ switches per DSB-induced cell; this is 5,000-fold higher than the natural rate of antigenic variation, estimated at approximately 1×10^−5^ switches per cell, per generation [13]. As expected, analysis of 24 silent VSG^pro^ (expressing *VSG121*) and 25 silent VSG^down^ (expressing *VSGX*) DSB-survivors failed to reveal any activation of the silent *VSG221* gene triggered by a break within the silent BES (data not shown).

Drug-sensitivity assays confirmed that DSBs were generated in the majority of non-switched survivors from the VSG^pro^ and VSG^down^ active site strains; 22/22 and 18/23 of these non-switched survivors were drug-sensitive, indicating disruption of *RFP:PAC*, and *NPT* expression, respectively (see FIG. 2A). Among non-switched VSG^pro^ survivors, three displayed repair *via* MMEJ as described previously [21]. Based on a previous analysis [27], we speculated that a T_2_AG_3_-like sequence downstream of *VSG221* served as a telomere-seed in the majority of non-switched VSG^down^ survivors, allowing for repair by *de novo* telomere addition. This was confirmed using PCR assays (FIG. S2A–B) and also explains continued *NPT* expression in five of these clones. Taken together, our results confirm the generation of DSBs in non-switched survivors and show that these breaks often fail to trigger antigenic variation when adjacent to the BES promoter or the T_2_AG_3_-repeats.

We also used a series of PCR assays, as above (see FIG. S2A), to confirm that DSBs had been generated in survivors from the silent VSG^pro^ and VSG^down^ strains. From the VSG^pro^ strain, eight survivors (33%) lost both the promoter-adjacent *RFP:PAC* gene and the *VSG221* gene and nine (38%) lost only *RFP:PAC*; the remaining seven (29%) repaired within *RFP:PAC* (data not shown) *via* MMEJ [21]. From the VSG^down^ strain, 24 survivors (96%) retained a promoter-adjacent *RFP:PAC* gene, eleven (44%) retained *VSG221* and only five (20%) retained *NPT* (data not shown). These results illustrate, consistent with the cloning-efficiency data shown in Figure 2C, how DSBs at either end of a silent BES are well-tolerated, even if they result in loss or replacement of part or all of the BES.

### The Mechanism of Antigenic Variation Is Highly Dependent upon Subtelomeric Break Site

We next used our series of PCR assays (see FIG. S2A) to explore the DNA rearrangements associated with antigenic variation. Following a DSB adjacent to the 70-bp repeats (VSG^up^ strain), we found that *VSG221* was lost in all but one of the switched survivors (FIG. 3B, clone 15), while only one of these also lost *ESAG1* (FIG. 3B, clone 9; FIG. 3D). Thus, antigenic variation typically occurred through recombination within the 70-bp repeats following a break adjacent to these repeats, as reported previously [10]. The clone that lost *ESAG1* may have switched through subtelomere loss or replacement, while the clone that retained *VSG221* may have switched through telomere crossover or promoter inactivation.

In striking contrast, following a DSB adjacent to the telomeric repeats (VSG^down^ strain), eight (89%) of the switched survivors lost *ESAG1* (FIG. 3C; FIG. 3D); the only clone that retained *ESAG1* had lost *VSG221* indicating recombination within the 70-bp repeats (FIG. 3C). We, therefore, asked whether a distal reporter adjacent to the promoter remained intact and active in the *ESAG1*-negative survivors; we had inserted an *RFP:PAC*-cassette adjacent to the BES promoter (see FIG. 2A) to monitor BES loss in the active VSG^down^ strain because we had previously observed BES loss following a DSB at the silent VSG^down^ site [28]. The analysis revealed that all eight *ESAG1*-negative survivors were also RFP negative by fluorescence microscopy (see FIG. 4A) and all but one of these had lost the *RFP-PAC* gene (FIG. 4B, FIG. S2C). We conclude that, when the DSB was adjacent to the telomeric repeats, seven of nine switched clones lost or replaced the BES; one clone underwent recombination within the 70-bp repeats and retained *ESAG1* while another clone underwent recombination elsewhere within the BES and inactivated the promoter, thereby retaining *RFP-PAC*.

In the two survivors that switched following a DSB adjacent to the promoter (VSG^pro^ strain), the *RFP:PAC*, *ESAG1* and *VSG221* genes were lost in one while all of these genes were retained in the other (FIG. S2D). This indicated BES loss or replacement in the first clone and promoter inactivation in the second; *RFP:PAC* sequencing revealed repair by MMEJ [21] in this second clone. Thus, DSBs adjacent to the 70-bp repeats trigger recombination within the 70-bp repeats; DSBs adjacent to the telomeric repeats often fail to do so, resulting in loss or replacement of the entire BES in around 25% of survivors, and DSBs at the promoter only rarely bring about antigenic variation. We also show that a break can occasionally lead to promoter inactivation. Figure 4C shows several examples of switched clones expressing new VSGs.

### RAD51-Independent Antigenic Variation

**Figure 5:**
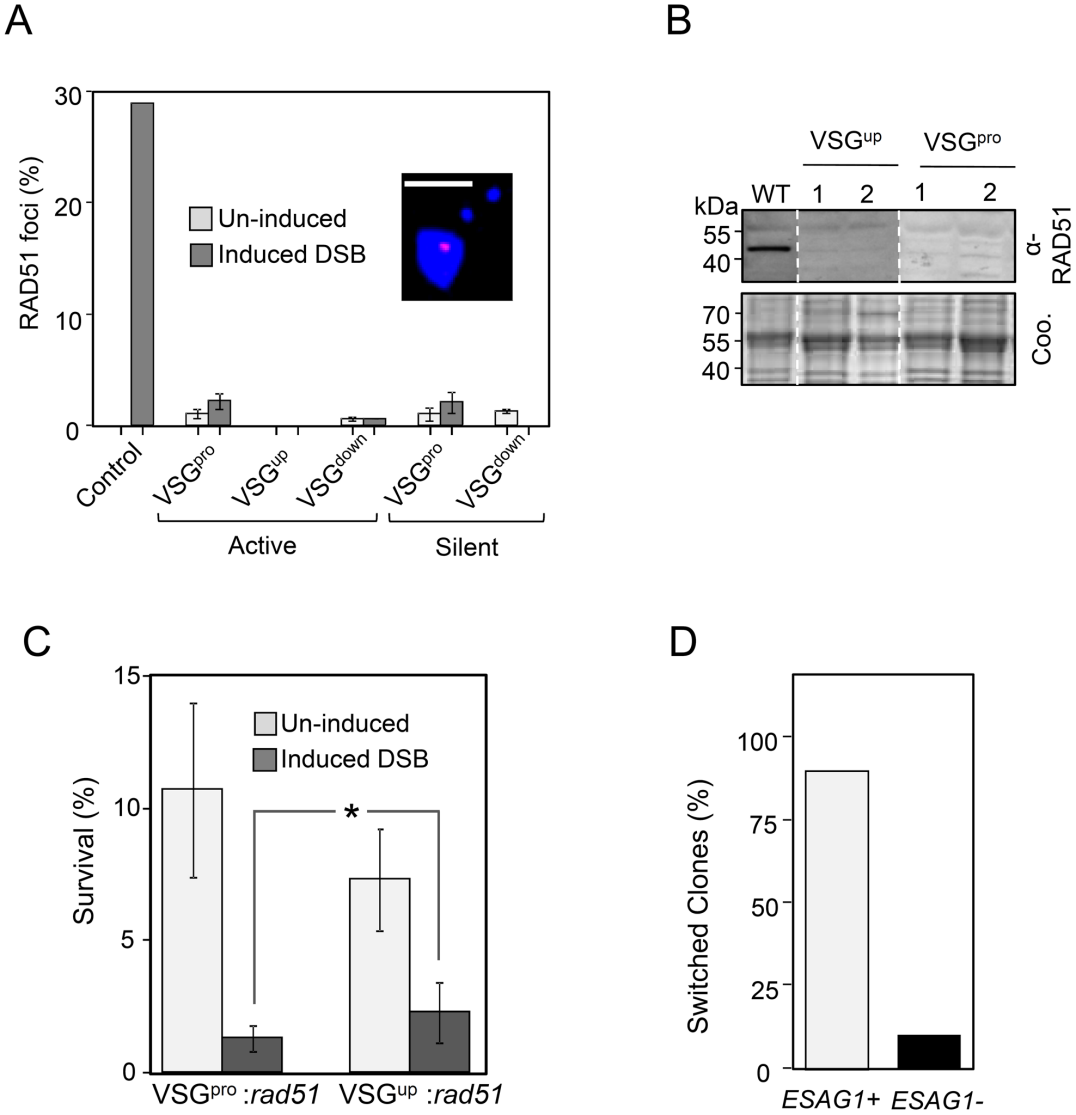
RAD51-dependent and independent BES repair and antigenic variation. (A) Nuclei with RAD51 foci were scored following a DSB at a chromosome-internal locus (Control) or at the active or silent BES. I-*Sce*I expression was induced for 12 h. n = 200 for each bar. Error bars for BES-break strains, SD. The inset shows a representative example of a nuclear RAD51 focus, red; DNA, blue; scale-bar, 5 µm. (B) Western blot analysis of *rad51* null strains, the Coomassie panels serve as loading controls. (C) Clonogenic assays. *rad51* null strains were distributed in 96-well plates under I-*Sce*I inducing conditions. Survivors were assessed after 7 days. Two independent clones were assayed in triplicate plates for each strain. A wild-type control displayed close to 100% survival (data not shown). Error bars, SD. *, *P*<0.05 based on Student's t-test. (D). PCR assays were used to check for the presence of the *ESAG1* gene in VSG^up^ *rad51*-null survivors following I-*Sce*I induction (n = 20).

*VSG* recombination and antigenic variation in *T. brucei* can occur *via* RAD51-dependent or RAD51-independent mechanisms [29]. These are most likely based on homologous strand-exchange and MMEJ, respectively [21]. Although *T. brucei* RAD51 forms sub-nuclear foci following induction of DSBs at a chromosome-internal locus [20], no significant increase in the proportion of cells with RAD51 foci was observed following induction of DSBs at BESs (FIG. 5A). This may reflect failure to accumulate RAD51 or a reduced dosage of accumulated RAD51. We therefore used a *rad51* gene knockout approach in both the active VSG^pro^ and VSG^up^ backgrounds (FIG. 5B). Clonogenic assays, using *rad51* null strains, allowed us to quantify the contribution of RAD51 to subtelomeric DSB repair and antigenic variation. The cloning efficiency of *rad51*-null strains is only approximately 10% prior to I-*Sce*I induction, indicating a major defect in DNA repair in the absence of RAD51 (FIG. 5C). Following I-*Sce*I induction, cloning efficiency was reduced further by approximately 90% (VSG^pro^:*rad51* strain) or 70% (VSG^up^:*rad51* strain). By comparing cloning efficiency in the VSG^up^-*rad51* strain and the VSG^up^-*RAD51* strain (2.3% v 6.2%; compare FIG. 5C and FIG. 2C), we see that approximately 40% of VSG^up^ survivors are RAD51-independent. Based on significantly higher DSB-survival in the VSG^up^:*rad51* strain compared to the VSG^pro^:*rad51* strain (FIG. 5C), we tentatively suggest more efficient RAD51-independent repair in the VSG^up^ strain. Among a panel of VSG^up^ *:rad51* survivors, twenty (91%) had undergone VSG switching, as determined by VSG221 immunofluorescence assay and, similar to the results in a *RAD51* background, all of these had lost *VSG221* and only two had lost *ESAG1* (FIG. 5D). These results indicated RAD51-independent recombination within the 70-bp repeats. Thus, RAD51-independent (likely MMEJ-based) recombination makes an important contribution to antigenic variation and we suggest that it is more efficient within 70-bp repeat sequences than within non-repetitive sequences.

### DNA Double-Strand Breaks Trigger DNA Resection at Active and Silent BESs

**Figure 6:**
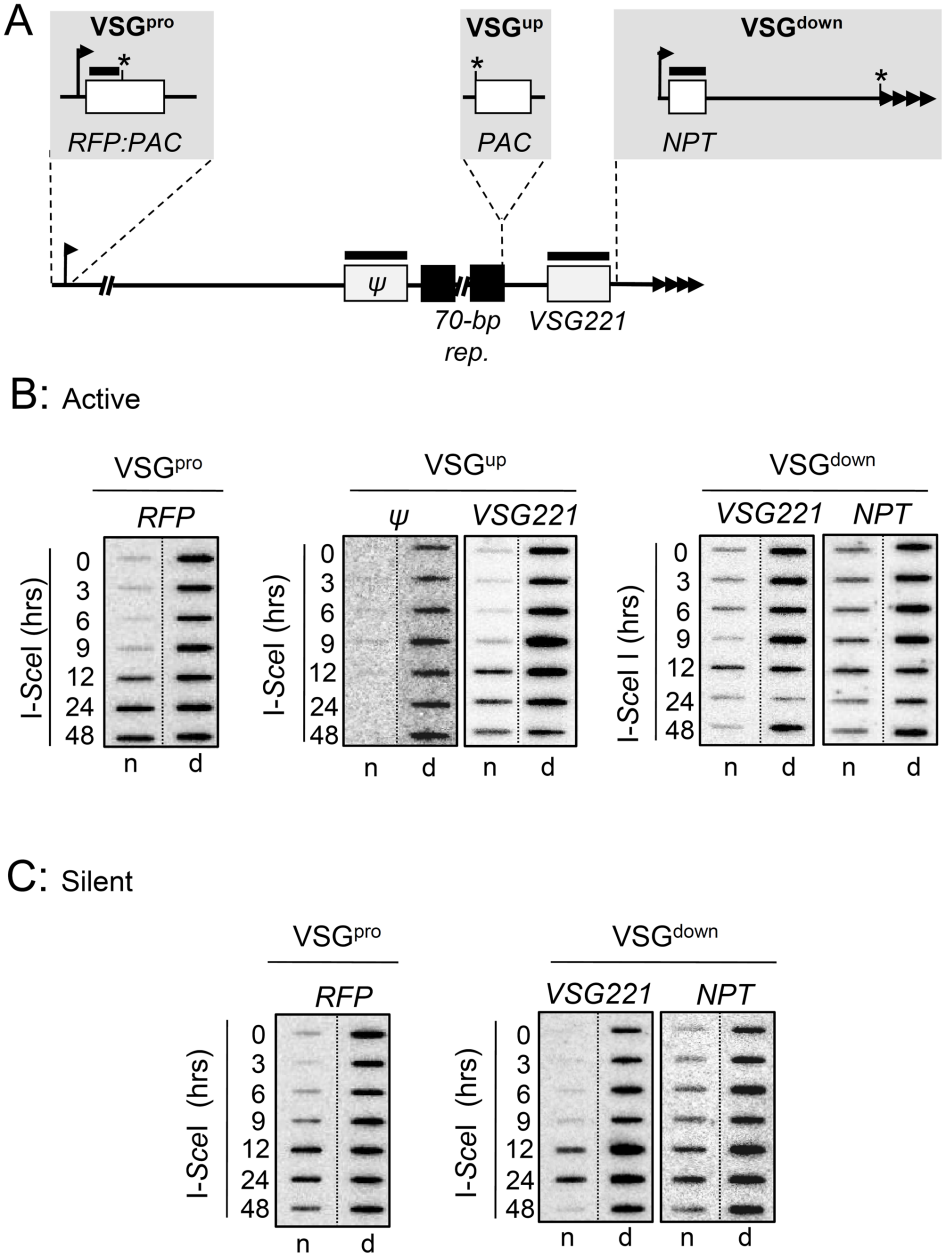
DNA double-strand breaks initiate DNA resection at active and silent *VSG* expression sites. (A) The schematic indicates the location of the I-*Sce*I cleavage sites (*) and probes (bars). Other details as in Figure 2A. (B) Assays in strains with an active *VSG221* BES. Genomic DNA samples were extracted at the times indicated following I-*Sce*I induction, and ssDNA was monitored on slot-blots. Ninety percent of each sample was ‘native’ (n) and the remaining 10% denatured (d) as a ‘loading’ control. (C) Assays in strains with a silent *VSG221* BES. Other details as in B above.

A common DSB response is local DNA resection, involving degradation of the 5′ strand of dsDNA to generate ssDNA with a 3′ end. The resulting ssDNA serves as a substrate for the assembly of DNA repair and recombination factors [30]. We used a series of slot-blot assays (FIG. 6A) to monitor DNA resection following induced DSBs. In these assays, specific probes are used to detect signals on native DNA and denatured DNA in parallel, revealing the presence of single-stranded regions or the sum of both single-stranded and double-stranded regions, respectively. In all strains analyzed, with breaks at active (FIG. 6B) and silent BESs (FIG. 6C), we detected local resection, typically peaking 12 h after meganuclease induction. The signal is reduced for the active VSG^down^ strain, but this may be due to the greater distance between the DSB and the regions probed for ssDNA, and also complete loss of the *VSG221* and *NPT* genes in some cells (see reduced signals in the ‘d’ columns). Thus, DNA resection is a common response to DSBs within a BES. We did note, however, failure to detect resection on the DSB-distal side of the 70-bp repeats in the active VSG^up^ strain (FIG. 6B; compare *Ψ* and *VSG221* probes). This suggested inefficient resection through the 70-bp repeats, either due to the rapid formation of recombination intermediates or some other property of the repeat-sequence itself. This is consistent with a role for the 70-bp repeats in facilitating *VSG* diversification by increasing the efficiency of recombination and also in serving as a ‘buffer’ that helps to protect the rest of the BES and the chromosome from the fragile end.

### Telomere-Repeat-Adjacent DNA Double-Strand Breaks Fail to Trigger a Cell-Cycle-Checkpoint

**Figure 7:**
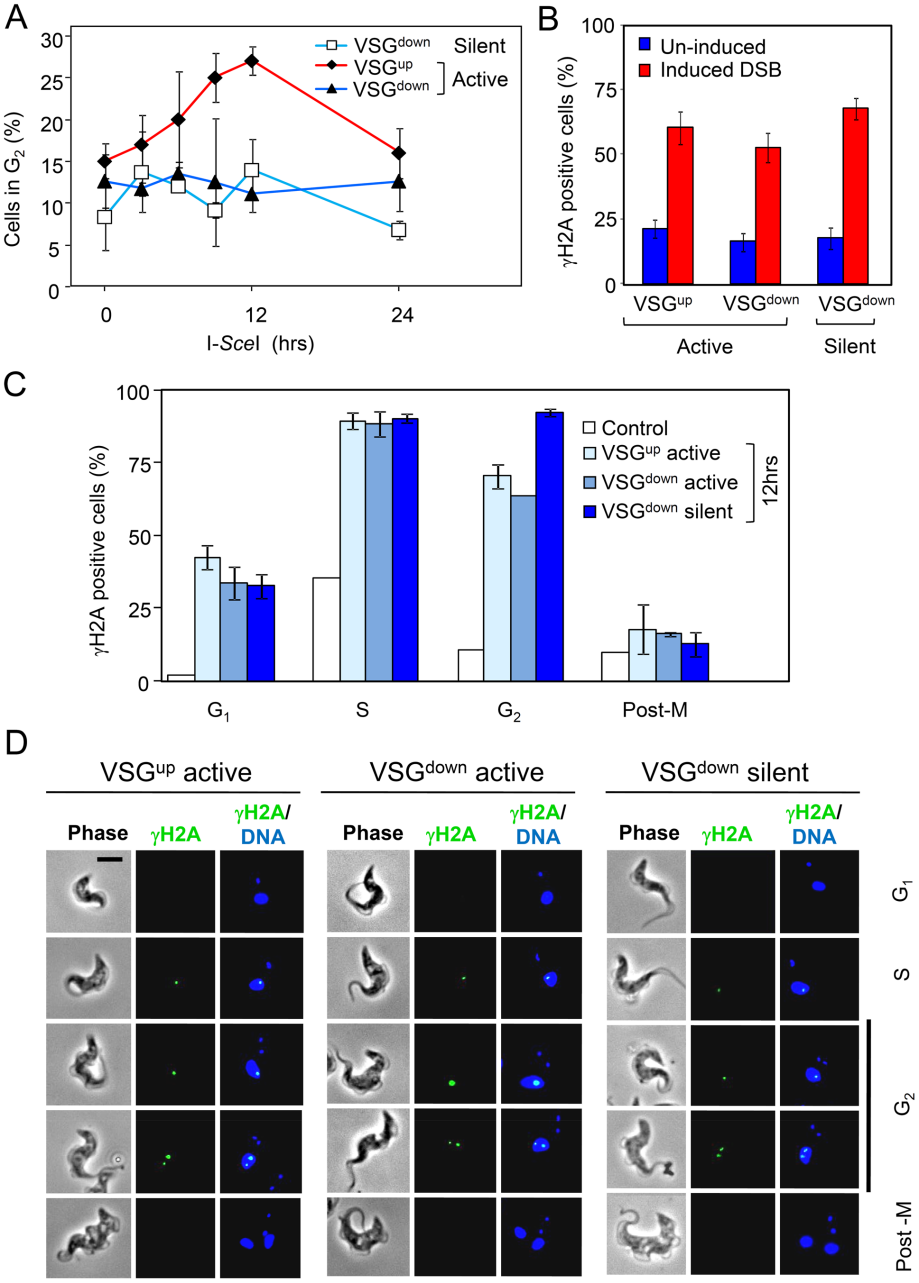
Distinct subtelomeric DNA double-strand break responses. (A) Cell-cycle phase was determined by DAPI-staining and defined by the number of nuclei (N) and kinetoplasts (K); G_2_, a single nucleus and two separate kinetoplasts (n = 200 at each time point). Error bars, SD. (B) γH2A accumulates at sub-nuclear foci in response to a DSB at a BES. Proportions of nuclei with foci were counted in uninduced (0 h) cells and 12 h after I-*Sce*I-induction (n = 200 at each time point). Error bars, SD. (C) γH2A foci are enriched in S-phase and G_2_. Cell-cycle phase was defined as above; G_1_, a single nucleus and a single rounded kinetoplast; S-phase, a single nucleus and an elongated kinetoplast; post-mitotic (post-M), two nuclei and two kinetoplasts (n = 100 for each bar). Error bars for BES-break strains, SD. Control; uninduced cells. (D) Immunofluorescence microscopy analysis of γH2A. Gallery of representative images showing cells with focal accumulation of γH2A during the cell-cycle 12 h after I-*Sce*I induction. Scale bar, 5 µm.

We previously reported continued cell cycle progression following *T. brucei* telomere deletion [28] and, in contrast, activation of a G_2_/M checkpoint in response to a DSB at a chromosome-internal locus [20]. We speculated that a severed DSB response [31] could explain failure to use the 70-bp repeats for recombination in the VSG^down^ strain. We used DAPI-stained nuclear and mitochondrial (kinetoplast) DNA as cytological markers to define position in the nuclear cell-cycle [32] and to examine cell cycle checkpoint responses; specifically, cells with a single nucleus and two separated kinetoplasts (1N2K) correspond to nuclear G_2_. A comparison of cells following DSBs in the silent VSG^down^ strain or in the active VSG^down^ or VSG^up^ strains, revealed an increased proportion of G_2_ cells only in the VSG^up^ strain (FIG. 7A). Thus, T_2_AG_3_ repeat-adjacent DSBs, in either silent or active BESs, fail to trigger the G_2_/M checkpoint. This may be analogous to the anticheckpoint mediated by telomere-repeat sequences in yeast [33]. This analysis also revealed a later accumulation of post-mitotic (2N2K) cells, between 24 and 48 h after I-*Sce*I induction, in all three strains with DBSs at the active BES (data not shown). Since VSG expression is required for progression to cytokinesis [25], later accumulation of post-mitotic cells supports the view that DSB responses interfere with local transcription [26] rather than transcription interfering with the DSB response.

Previously, it has not been possible to observe DNA damage and repair foci associated with BESs (see FIG. 5A). We recently described *T. brucei* γH2A, a phosphorylated form of histone H2A that accumulates at DNA repair foci in response to DNA damage [34]. Immunofluorescence microscopy was used to explore the subnuclear accumulation of γH2A foci in response to DSBs in the strains described above. Although telomere-adjacent breaks failed to trigger the G_2_/M checkpoint, we observed robust γH2A responses in all strains examined (FIG. 7B); I-*Sce*I induction increased the proportion of cells with γH2A foci from approximately 20%, representing naturally occurring DNA-damage, to >50%, representing additional BES-associated breaks. We next assessed the appearance of these γH2A foci during the cell-cycle. In all cases, foci were predominantly associated with the S- and G_2_-phases (FIG. 7C), as described previously for natural breaks and for chromosome-internal breaks [34]. Representative images are shown in Figure 7D and reveal indistinguishable foci in the three strains presented. Thus, we conclude that γH2A foci that form in response to telomere repeat-adjacent breaks fail to signal the G_2_/M checkpoint but are still efficiently disassembled prior to progression to mitosis. These results are consistent with a telomere-adjacent DNA damage response that is severed after DNA resection and γH2A focus assembly but prior to the G_2_/M checkpoint.

## Discussion

**Figure 8:**
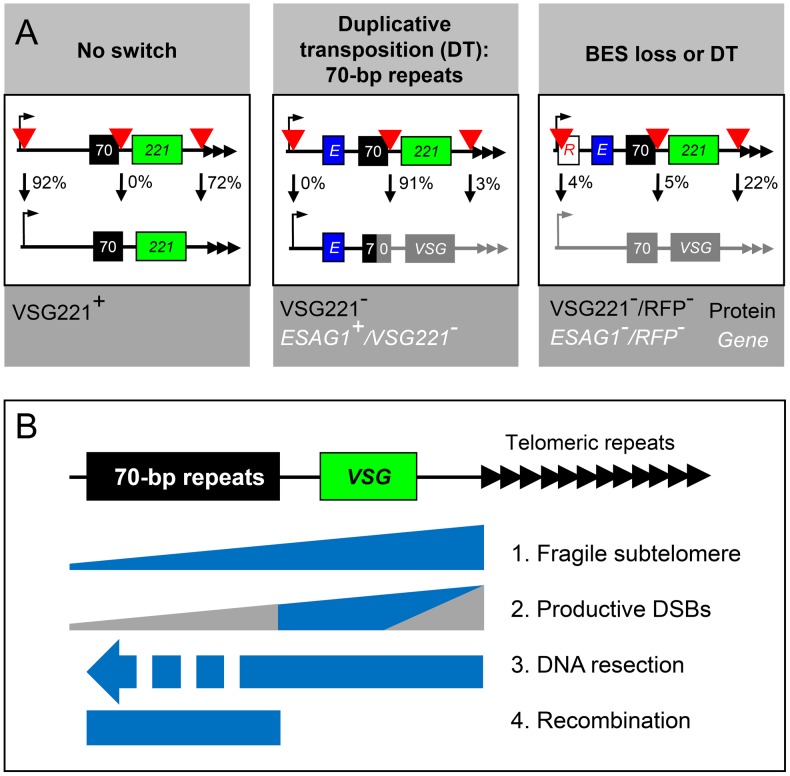
Summary of outputs and model for *VSG* replacement. (A) The schematic shows the active BES with the relevant markers. Left, DNA repair leaves the active *VSG* intact; middle, 70-bp repeat recombination replaces the *VSG* through duplicative transposition; right, loss or replacement of the entire BES. Arrow, BES promoter; *R*, *RFP:PAC*; *E*, *ESAG1*; 70, 70-bp repeats; *221*, *VSG221*; black arrowheads, T_2_AG_3_-repeats. Red arrowheads, sites of induced DSBs in VSG^pro^, VSG^up^ and VSG^down^ strains. Percentages of survivors that displayed each outcome are indicated. (B) Model to explain antigenic variation via subtelomere fragility and 70-bp repeat recombination at the active BES. 1. Breaks may be more frequent closer to the telomeric-repeats (blue wedge). 2. Breaks adjacent to the telomeric-repeats initiate a distinct DNA damage response and typically fail to use the 70-bp repeats for recombination, while breaks within the 70-bp repeats would be expected to be repaired by single-strand annealing (grey wedges). The remaining breaks (blue region) are productive, in that they initiate resection that progresses towards the 70-bp repeats from the telomeric side of these repeats and also allow for recombination within these repeats. 3. A DSB triggers resection (blue bar) and reveals ssDNA, which initiates a search for recombination templates. Recombination in the 70-bp repeats would terminate further resection in this region (dashed blue bar). 4. The 70-bp repeats provide a template for the initiation of (micro)homologous recombination and duplicative transposition (blue bar). We propose that recombination is favored within these repeats because they are highly repetitive and widely dispersed.

We have shown that the subtelomere, within a *VSG* expression site in *T. brucei*, is fragile, displaying more breaks than seen at a chromosome-internal locus and also some evidence of increased fragility closer to the telomeric repeats. We also show that the location of a subtelomeric break has a major impact on probability and mechanism of antigenic variation. We demonstrate subtelomeric DSB responses that include DNA resection, histone modification and checkpoint activation. Notably, breaks immediately adjacent to the telomere fail to trigger a checkpoint, possibly promoting BES loss or replacement. The consequences in terms of antigenic variation, following DSBs at three distinct sites within an active *VSG* BES, are summarized in Figure 8A. In Figure 8B, we present a model, based on our findings, to explain how repetitive sequences flanking *VSG* genes cooperate to drive antigenic variation and host immune evasion.

While DSBs were estimated in ∼1% of cells in the only other report of meganuclease-induced breaks at the active BES [10], we report induction of DSBs in close to 100% of cells. The efficiency, specificity and temporal constraint of meganuclease cleavage achieved here allowed us to apply a quantitative approach to dissecting subtelomeric DSB responses and the consequences for antigenic variation. The ability to induce a defined break, in almost every cell in the population, also facilitated genetic dissection of DSB repair, and allowed for analysis both microscopically and using physical monitoring techniques. Accordingly, we assessed the contribution of RAD51 and monitored DNA-damage responses, including assembly of subnuclear repair foci and DNA resection. Importantly, we have been able to study all DSB-survivors, those that undergo antigenic variation, and those that repair the subtelomere without switching *VSG* expression; as far as we are aware, the first time this has been achieved. Previous studies typically relied upon positive or negative selection protocols, involving activation or inactivation of a *VSG*-linked drug selectable marker or the *VSG* itself. These approaches yielded only cells that had undergone antigenic variation, made it difficult to define individual members of a panel of switched clones as independent and potentially introduced bias in terms of the relative contribution of each switching mechanism.

Our analyses provide quantitative insights into the relationship between DSBs, subtelomeric recombination mechanisms and antigenic variation mechanisms in *T. brucei*. We propose a model whereby both sets of *VSG*-flanking repeats, telomeric and 70-bp, cooperate to bring about antigenic variation (FIG. 8B); fragility within the subtelomeric region increases the frequency of DSBs, the triggers for antigenic variation, while the 70-bp repeats, in association with archival *VSG*-associated repeats, facilitate recombination and replacement of the active *VSG*.

### Fragile Subtelomeres Trigger Antigenic Variation in *T. brucei*

Our survey of the *VSG221* locus suggests that natural DSBs could be more frequent closer to the telomeric T_2_AG_3_-repeats. Indeed, the subtelomeric regions of a number of cell types have been shown to be fragile and prone to frequent breakage [35]. For example, human subtelomeres are recombination hot-spots [36] and mammalian telomeres are fragile sites [37]. Subtelomeres are also unstable in the malaria parasite, *P. falciparum*, and undergo frequent breakage and repair [38]. Our findings now indicate that subtelomeres are also fragile in African trypanosomes.

So why are subtelomeres prone to breaks? Our results indicate fragility independent of transcription, implicating DNA replication as the source of these breaks. Indeed, replication stress and fork collapse during S-phase is likely a major source of DSBs in all eukaryotes [39]. Subtelomeric DNA, due to secondary structure or local chromatin structure, could be particularly prone to replication stress, making replication forks more likely to stall and collapse. In this regard, it is notable that an I-*Sce*I site embedded within telomeric repeats at the active BES was not cleaved following I-*Sce*I induction *in vivo* (FIG. S1), suggesting inaccessible chromatin associated with tracts of T_2_AG_3_-repeats. The apparent transition from (I-*Sce*I) accessible to inaccessible chromatin at the T_2_AG_3_-repeat junction could present a challenge for the replication machinery to negotiate.

It has been proposed that short telomeres at the active BES are prone to breaks that increase the rate of antigenic variation [40], [41]. This cannot explain high numbers of breaks detected in our LM-PCR assays, however, since the active *VSG221*-associated T_2_AG_3_-tracts are in excess of 5-kbp in all of the strains used here [27, also see FIG. 2B and ] FIG. S1. The 70-bp repeats have also been proposed to be the source of frequent breaks that trigger antigenic variation [10]. Deletion of the 70-bp repeat tract at the active BES demonstrated a role for these tracts in duplicative transposition [10], [19], but these studies did not distinguish between roles in triggering breaks or in subsequent recombination. We suggest that breaks within the 70-bp repeats, or between two blocks of 70-bp repeats [10], would generate effective substrates for single-strand annealing [42], a recombination pathway which would generate a ‘repeat’ deletion, rather than lead to *VSG* replacement. Breaks on the *VSG*- and telomere-proximal side of the 70-bp repeats, on the other hand, clearly do trigger antigenic variation [10; this study].

### Subtelomeric Break Site Determines Probability and Mechanism of Antigenic Variation

We show that the probability of antigenic variation is highly dependent upon the site of the subtelomeric DSB at the active BES. These breaks are not well-tolerated, however, and cell death is a common outcome. Even successful repair within the active BES commonly fails to bring about antigenic variation following breaks at certain sites. These findings are consistent with the high rate of natural DSBs that we observe at the active BES, relative to antigenic variation, and suggest that cells often die or fail to switch following these natural DSBs. Lesions at the active BES are probably typically lethal because VSG expression is compromised, while genes within silent BESs are dispensable and loss of these genes is tolerated.

Our results also show that the site of a subtelomeric break has a major impact on the mechanism of antigenic variation. Subtelomeric breaks on either side of the active *VSG* can trigger antigenic variation but a DSB adjacent to the telomeric repeats is substantially less efficient in this regard. It is notable that a DSB within the BES can also trigger promoter inactivation. One switched survivor from the VSG^pro^ strain underwent MMEJ and inactivated the promoter and another from the VSG^down^ strain inactivated the promoter and lost part of the BES. These are similar to *in-situ* switching events and may explain RAD51-dependent *in-situ* switching as reported previously [22]. Thus, *in situ* switching can be triggered by DSB-repair that does not substantially alter the sequence of the BES.

*T. brucei* TOPO3α suppresses RAD51-dependent crossovers and recombination beyond the 70-bp repeats within the BES, thereby favoring recombination within these repeats [13]. We find, consistent with previous studies [13], [22], that antigenic variation associated with 70-bp repeat-recombination involves both RAD51-dependent and independent pathways. Notably, however, our results suggest a higher rate of RAD51-independent recombination within the 70-bp repeats than observed in the BES promoter region. MMEJ is RAD51-independent and we suggest that this repair mechanism is more efficient within 70-bp repeat sequences, due to the relative abundance of potential ‘microhomologies’. Thus, recombination followed by Break-Induced Replication to the chromosome end and replacement of the active *VSG* could be initiated by microhomology.

### Checkpoint Bypass and Subtelomere Loss

Our data do not reveal differences in the DNA damage response due to BES transcription in *T. brucei*. Rather, they reveal a different response due to telomere-repeat proximity. We show that subtelomeric breaks trigger γH2A focus formation and DNA resection. The increase in γH2A foci in response to DSBs allowed us, for the first time, to visualize repair sites associated with *VSG* recombination. Notably, γH2A focus formation is associated with a G_2_/M cell-cycle checkpoint following DSBs upstream of the active *VSG* but not following breaks immediately adjacent to the telomeric repeats. These latter cells also failed to use the 70-bp repeats for recombination and, instead, underwent antigenic variation *via* BES loss or replacement. Failure to trigger this checkpoint following telomere-repeat-adjacent breaks was independent of the transcription status of the BES.

Telomere-associated proteins are known to repress the DNA damage response [43]. In *Schizosaccharomyces pombe*, a telomeric DSB-response is severed due to the absence of epigenetic marks required for cell-cycle arrest [31], and telomeric repeats also suppress the checkpoint response in *Saccharomyces cerevisiae* [33]. This anticheckpoint effect is thought to prevent the fusion of linear chromosomes. We propose the operation of a similar anticheckpoint in *T. brucei*. Our results suggest a checkpoint bypass mechanism when the break is adjacent to the telomeric repeats and the G_2_/M checkpoint may be required for efficient participation of the 70-bp repeats in recombination. Natural breaks adjacent to the telomeric repeats may similarly explain previous reports of BES loss or replacement [12], [13], [14], [15].

### Concluding Remarks

DNA DSBs are triggers for antigenic variation. Here, we probe DSB responses, BES recombination pathways and mechanisms of antigenic variation. First, we show that subtelomeres are fragile; thereby generating the DNA breaks that trigger antigenic variation. We then demonstrate *VSG* replacement and BES loss in response to distinct subtelomeric breaks, and also provide evidence for *in situ* switching as a response to subtelomeric DSBs. It is 70-bp repeat recombination that makes the major contribution to antigenic variation because most archival *VSGs* are flanked by these repeats and use them for gene-conversion. We suggest that breaks between the telomeric and 70-bp repeats trigger this pathway. What follows is a DNA damage response that includes DNA resection, histone modification and, depending upon the site of the break, a G_2_/M checkpoint. Formation of 70-bp repeat ssDNA then promotes interaction with similar templates elsewhere in the genome; these repeats may be favored substrates for recombination simply because they are highly repetitive. Recombination is then either RAD51-dependent or RAD51-independent; most probably MMEJ-based in this latter case. In conclusion, we provide novel insight into the triggers, associated DNA damage responses and mechanisms of antigenic variation in African trypanosomes. Our findings may also be relevant to subtelomeric gene rearrangements in human cells and to immune evasion mechanisms in other pathogenic protists, fungi and bacteria, such as *Plasmodium* sp., *Pneumocystis* sp. and *Borrelia* sp., respectively [44].

## Materials and Methods

### *T. brucei* Strains

*T. brucei* Lister 427 cells were grown and genetically manipulated as described [28]. The strain referred to here as VSG^down^-silent was described previously [28]. Puromycin or G418 selection (2 µg/ml) were used to ensure that the *VSG221* BES remained active prior to I-*Sce*I induction. I-*Sce*I was induced using tetracycline (Tet) at 1 µg/ml (Sigma). For clonogenic assays, a mean of 0.3 to 50 cells per well were seeded in 96-well plates with or without Tet. Survivors were assessed microscopically after 5–7 days. All clones analyzed were from plates with <30% positive wells. Repaired survivors were scored for puromycin sensitivity at 1 µg/ml. DSB-survivors that displayed >99% VSG221 positive cells, as determined by immunofluorescence analysis, were scored as non-switched, while survivors that displayed >98% VSG221 negative cells were scored as switched. Proportion of 1N2K cells and cells with γH2A repair foci were counted by two of us to generate mean values ± SD.

### Plasmid Construction

The BES promoter-targeting constructs, pESP-*RFP:PAC*, pESP*-R^S^P* and pESPi*-R^S^P* were derived from pESPi*RFP:PAC* [28]. Briefly, the tetracycline-operator was removed from pESPi*RFP:PAC* to derive pESP-*RFP:PAC* and an I-*Sce*I site was added to derive pESP*-R^S^P*. To insert an I-*Sce*I site at the *Not*I site between the *RFP* and *PAC* genes, ‘I-*Sce*I’ primers were annealed and ligated to give pESP*-R^S^P*. The *R^S^P* cassette replaced *RFP-PAC* in pESPi*RFP-PAC* to give pESPi-*R^S^P*. Transfections with *Sac*I-*Kpn*I digests of pESP*-R^S^P* or piESP*-R^S^P* were used to generate VSG^pro^ active and silent strains, respectively. The ES-70 cassette was assembled using primers containing the I-*Sce*I site and targeting fragments to amplify the *PAC* resistance cassette. The PCR product was transfected to generate VSG^up^ strains. pTMF-Sce [28] was digested with *Sma*I and transfected to generate VSG^down^ active strains. To generate the pTMF^Em^ construct SceHexU/SceHexL primers were annealed and ligated to *Spe*I/*Pst*I digested pTelo1 (pBluescript with sixteen T_2_AG_3_-repeats at the MCS). pTMF^Em^ was digested with *Sma*I and transfected to generate VSG^telo^ strains. *RAD51* gene disruption targets were amplified by PCR from *T. brucei* genomic DNA, using Phusion high-fidelity DNA polymerase (New England Biolabs). The targets were assembled such that they flanked *BSD* or *NPT* selectable markers. Both constructs were digested with *Acc*651 and *Not*I prior to transfection. Details of primers/oligonucleotides are available on request.

### DNA Analysis

Ligation-mediated PCR (LM-PCR) was carried out as described [10]. Briefly, DNA DSBs were detected by in-gel blunt-end linker ligation and PCR. The BES locus-specific primers were: LMPCRi (tagcagaatgcaacgtcga), LMPCRii (ttggcgactataacggctg) and LMPCRiii (ggcgttaccaagcttgttga). Slot blots for the detection of ssDNA were carried out as described [20]. Southern blotting and sequencing were carried out according to standard protocols [45]. *RFP*, *PAC*, *ESAG1* [13], *VSG221* and telomere-repeat-specific primers were used for the PCR assays. Other details of oligonucleotides are available on request.

### Protein Analysis

Extracts of total cell protein were separated on SDS-polyacrylamide gels and stained with Coomassie-blue or subjected to western blotting using standard protocols [45]. We used rabbit anti-VSG221, rabbit anti-RAD51 [23] and an ECL+ kit (GE Healthcare). For immunofluorescence microscopy, cells were labeled using a standard protocol with rabbit anti-VSG221 rabbit anti-γH2A [34] or mouse anti-Myc (Source Bioscience), and fluorescein or rhodamine-conjugated goat anti-rabbit or anti-mouse secondary antibodies (Thermo Scientific Pierce Antibodies). RFP was detected directly. Cells were mounted in VectaShield (Vector Laboratories) containing 4, 6-diamidino-2-phenylindole (DAPI). Images were captured on an Eclipse E600 microscope (Nikon) using a Coolsnap FX (Photometrics) charged coupled device camera and processed in Metamorph 5.4 (Photometrics).

## Supporting Information

Figure S1 **Failure to generate a DSB when the I-** ***Sce*** **I site is embedded within telomeric-repeat sequence.** An I-*Sce*I cleavage site (*) was engineered such that it was embedded within T_2_AG_3_-repeat sequence at the active BES, as indicated in the upper panel. Genomic DNA from this VSG^telo^ strain, following I-*Sce*I induction, was digested with *Hpa*I. The probe used for Southern blotting (lower panel) was an *NPT* fragment. I-*Sce*I induction failed to cleave the site, as revealed by persistence of the terminal restriction fragment. A plasmid control was digested with *Hpa*I plus I-*Sce*I and the presence of the I-*Sce*I site was also confirmed in *T. brucei* genomic DNA (data not shown). The ethidium bromide (EtBr) stained gel shows loading. Other details as in Figure 2A–B.
(PDF)

Figure S2 **BES PCR assays.** (A) The schematic map indicates the location of primers used for the BES PCR assays. Other details as in Figure 2A. (B) The PCR assays demonstrate *RFP-PAC* and *VSG221* gene status, and include an assay for *de novo* telomere healing close to the *VSG221* gene in survivors from VSG^pro^-silent BES cells, following I-*Sce*I-mediated cleavage. +, positive control. (C) The PCR assay demonstrates *RFP-PAC* gene status following I-*Sce*I-mediated cleavage in switched survivors from VSG^down^-active BES cells (see FIG. 4B). (D) The PCR assays demonstrate *RFP-PAC*, *ESAG1* and *VSG221* gene status following I-*Sce*I-mediated cleavage in switched survivors from VSG^pro^-active BES cells. +, positive control.
(PDF)
