# Supplementary material for: DNA Break Site at Fragile Subtelomeres Determines Probability and Mechanism of Antigenic Variation in African Trypanosomes
Source: PLoS Pathog. 2013 Mar 28;9(3):e1003260. doi: 10.1371/journal.ppat.1003260 (PMC3610638; doi:10.1371/journal.ppat.1003260)
Supplement: Figure S1 — Failure to generate a DSB when the I- Sce I site is embedded within telomeric-repeat sequence. An I-SceI cleavage site (*) was engineered such that it was embedded within T2AG3-repeat sequence at the active BES, as indicated in the upper panel. Genomic DNA from this VSGtelo strain, following I-SceI induction, was digested with HpaI. The probe used for Southern blotting (lower panel) was an NPT fragment. I-SceI induction failed to cleave the site, as revealed by persistence of the terminal restriction fragment. A plasmid control was digested with HpaI plus I-SceI and the presence of the I-SceI site was also confirmed in T. brucei genomic DNA (data not shown). The ethidium bromide (EtBr) stained gel shows loading. Other details as in Figure 2A–B. (PDF) [file ppat.1003260.s001.pdf]

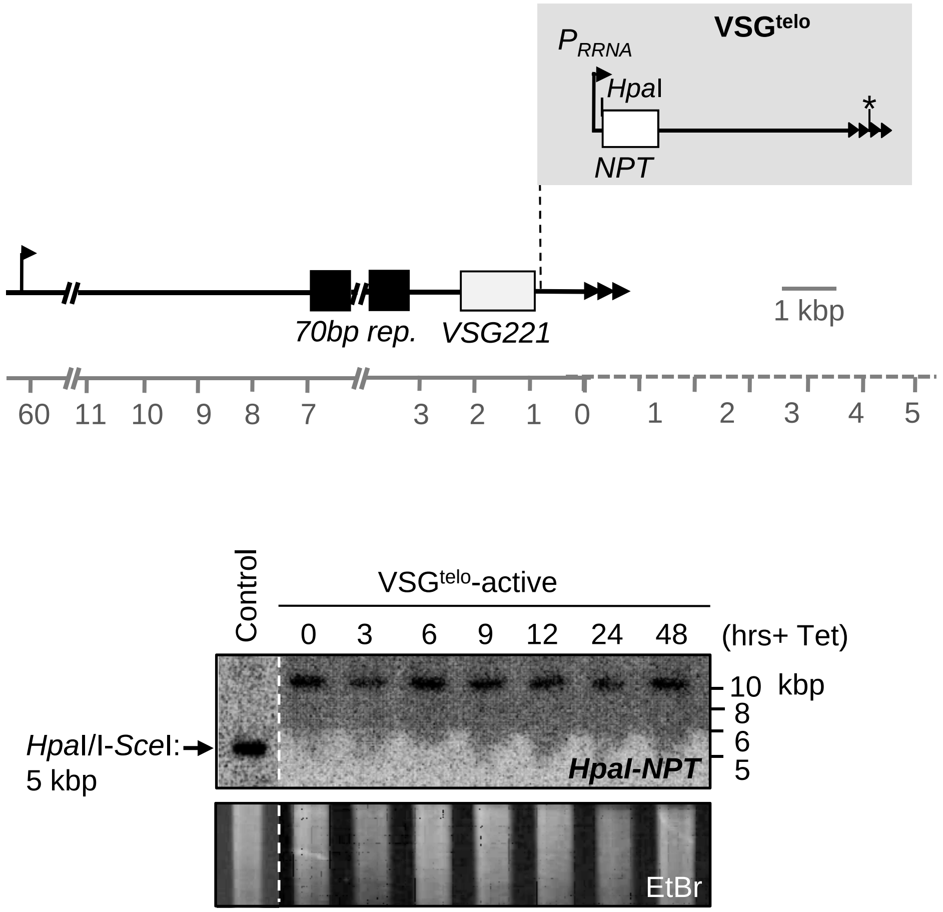

Figure S1 Failure to generate a DSB when the I-*Sce*I site is embedded within telomeric-repeat sequence. An I-*Sce*I cleavage site (*) was engineered such that it was embedded within T_2AG_3-repeat sequence at the active BES, as indicated in the upper panel. Genomic DNA from this VSG^{telo} strain, following I-*Sce*I induction, was digested with *Hpa*I. The probe used for Southern blotting (lower panel) was an *NPT* fragment. I-*Sce*I induction failed to cleave the site, as revealed by persistence of the terminal restriction fragment. A plasmid control was digested with *Hpa*I plus I-*Sce*I and the presence of the I-*Sce*I site was also confirmed in *T. brucei* genomic DNA (data not shown). The ethidium bromide (EtBr) stained gel shows loading. Other details as in Figure 2A-B.
