# Supplementary material for: DNA Break Site at Fragile Subtelomeres Determines Probability and Mechanism of Antigenic Variation in African Trypanosomes
Source: PLoS Pathog. 2013 Mar 28;9(3):e1003260. doi: 10.1371/journal.ppat.1003260 (PMC3610638; doi:10.1371/journal.ppat.1003260)
Supplement: Figure S2 — BES PCR assays. (A) The schematic map indicates the location of primers used for the BES PCR assays. Other details as in Figure 2A. (B) The PCR assays demonstrate RFP-PAC and VSG221 gene status, and include an assay for de novo telomere healing close to the VSG221 gene in survivors from VSGpro-silent BES cells, following I-SceI-mediated cleavage. +, positive control. (C) The PCR assay demonstrates RFP-PAC gene status following I-SceI-mediated cleavage in switched survivors from VSGdown-active BES cells (see FIG. 4B). (D) The PCR assays demonstrate RFP-PAC, ESAG1 and VSG221 gene status following I-SceI-mediated cleavage in switched survivors from VSGpro-active BES cells. +, positive control. (PDF) [file ppat.1003260.s002.pdf]

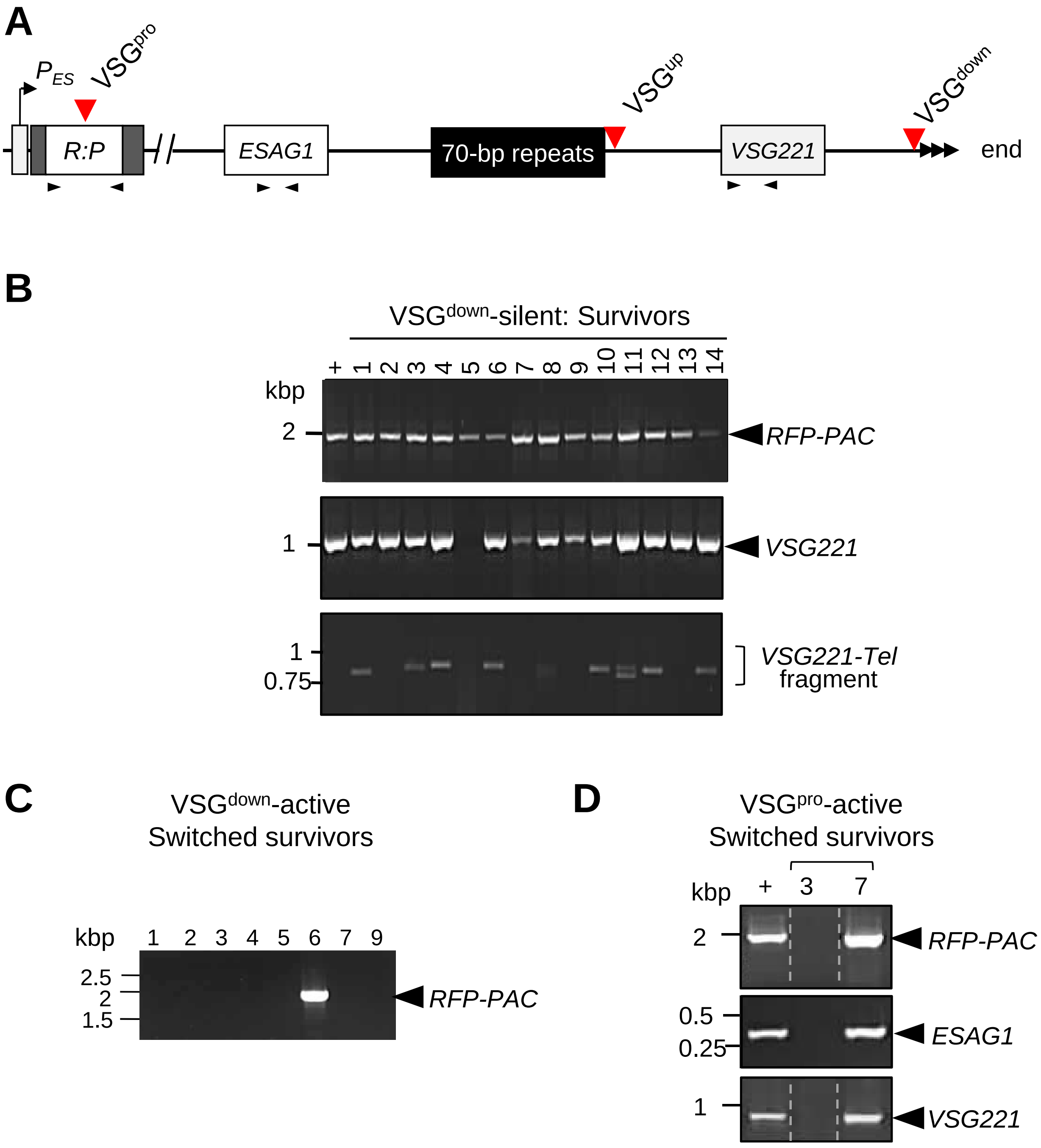

Figure S2. BES PCR assays. (A) The schematic map indicates the location of primers used for the BES PCR assays. Other details as in Figure 2A. (B) The PCR assays demonstrate *RFP-PAC* and *VSG221* gene status, and include an assay for *de novo* telomere healing close to the *VSG221* gene in survivors from VSG^{pro}-silent BES cells, following I-*Sce*I-mediated cleavage. +, positive control. (C) The PCR assay demonstrates *RFP-PAC* gene status following I-*Sce*I-mediated cleavage in switched survivors from VSG^{down}-active BES cells (see FIG. 4B). (D) The PCR assays demonstrate *RFP-PAC*, *ESAG1* and *VSG221* gene status following I-*Sce*I-mediated cleavage in switched survivors from VSG^{pro}-active BES cells. +, positive control.
